# Frykman VIII Fracture Secondary to Falling Onto an Outstretched Hand (FOOSH)

**DOI:** 10.7759/cureus.50641

**Published:** 2023-12-16

**Authors:** Vedha Dande, Dwayne D'Souza, Rohan Mangal, Anjali R Daniel, Latha Ganti

**Affiliations:** 1 Biomedical Sciences, University of Central Florida, Orlando, USA; 2 Emergency Medicine and Orthopedics, Vanderbilt University Medical Center, Nashville, USA; 3 Medicine, University of Miami Miller School of Medicine, Miami, USA; 4 Biology, Emory University, Atlanta, USA; 5 Medical Sciences, The Warren Alpert Medical School of Brown University, Providence, USA; 6 Emergency Medicine and Neurology, University of Central Florida College of Medicine, Orlando, USA

**Keywords:** fall on an outstretched hand, orthopedic trauma, radius fracture classification, distal radius fracture, frykman viii fracture

## Abstract

This is the case of a 41-year-old woman who presented with pain in the wrist after a fall from her bicycle, after which she tried to block her fall by outstretching her hand. She sustained a Frykman VIII fracture, with a fracture of the distal radius and ulnar styloid. Her labs were unremarkable, and she had no previous medical history. Her case was an example of a classic fracture due to FOOSH (falling onto an outstretched hand) which can easily be avoided by learning how to break falls properly. The patient’s symptoms, diagnosis, treatment, and ways to avoid this injury are presented.

## Introduction

The Frykman classification of distal radial fractures is based on the anteroposterior view of plain radiographs' appearance of the fracture [1]. There are several eponymous descriptions of distal radius fractures. A Colles fracture is a distal radius fracture with dorsal comminution, dorsal angulation, dorsal displacement, radial shortening, and an associated ulnar styloid fracture [2]. A Smith fracture, sometimes termed a “reverse Colles”, is a distal radius fracture with volar angulation. Both of these occur with falling onto an outstretched hand (FOOSH); a Colles fracture is seen when the patient falls on their outstretched hand in pronation, while a Smith fracture happens when the fall occurs and the wrist is in supination. A Barton fracture also occurs when the wrist is in pronation like a Colles fracture but involves a compression fracture resulting in shearing of the dorsal rim and associated distal radioulnar joint disruption and often subluxation/dislocation of the radiocarpal joint [3]. A Chauffeur fracture is an intra-articular oblique fracture of the radial styloid process [4]. The Frykman classification assesses the pattern of fractures, involvement of the radioulnar joint, and the presence of a distal ulnar fracture [5].

Ulnar styloid fractures occur in 55% of all distal radius fractures. In a study of 56 patients with both bones fractured, the most common Frykman types were II, VII, VIII, and V [6]. History shows that most fractures are expected to occur due to an evolutionary support mechanism while falling. When falling, most people use their hands to brace their fall and protect their head and neck and core from a direct impact, therefore, putting their wrist and the ulna styloid at risk for injury.

## Case presentation

**Figure 1:**
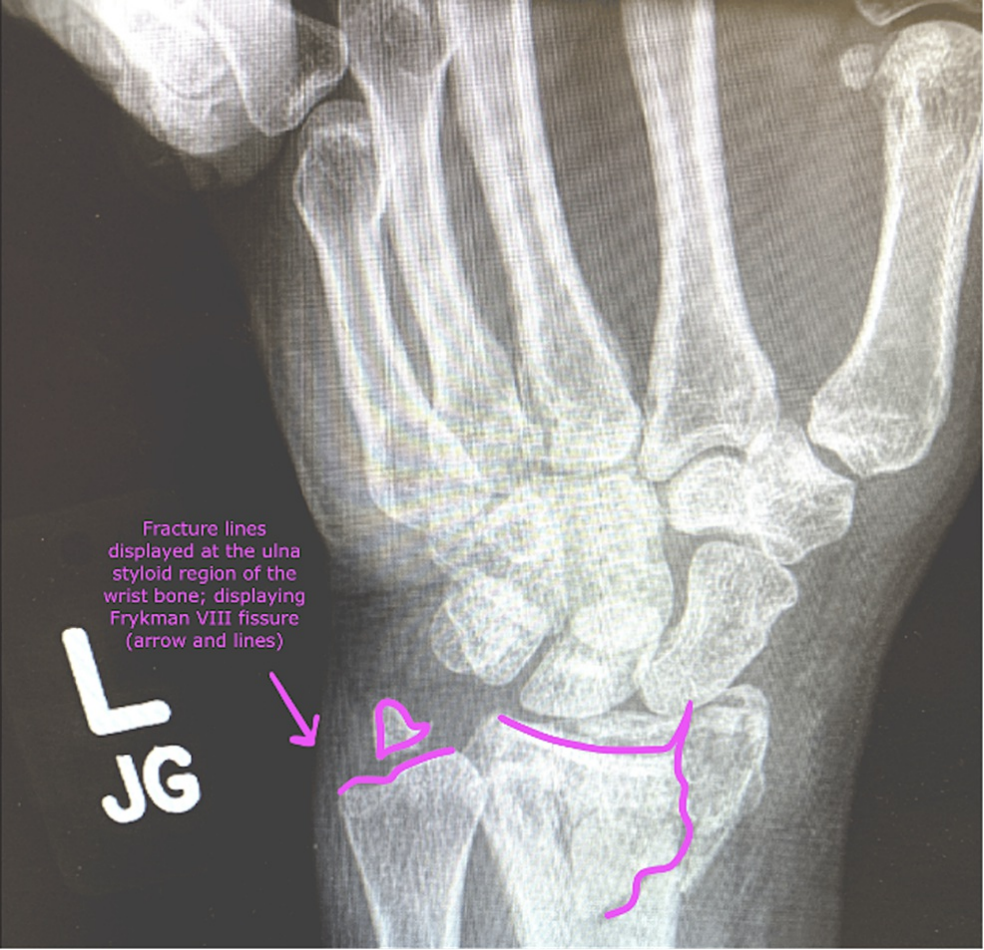
Anteroposterior view of the wrist demonstrating a Frykman VIII fracture

A 41-year-old woman presented to the emergency department (ED) with pain in her left wrist after a fall from her bicycle. She tried to break her fall by outstretching her hand. She landed on her hand and then tumbled over it to land on her right side, with her hand and wrist bearing the brunt of the force, followed by landing on her right shoulder. She did not hit her head or lose consciousness. Physical examination revealed a grossly deformed distal forearm. Her radial and ulnar pulses were intact, and there were no open wounds. She had “road rash” abrasions all over the right side of her shoulder, arms, and to a lesser degree on her legs. Vital signs revealed her to be afebrile, pulse of 104 beats per minute, respiratory rate of 22, blood pressure of 147/93 mmHg, and pulse oximetry 99% on room air. The patient was visibly anxious and reported a pain level of 10/10. The patient denied any medical or surgical history. She did endorse anxiety but said that she was not under any medical treatment for it. She denied any allergies and did not take any medications on a daily basis. Radiographs revealed a Fykman VIII fracture (Figure 1). 

## Discussion

Distal radius fractures are fairly common traumatic injuries presenting to the ED. Most of these fractures occur due to FOOSH and make up 25% of bone injuries for children, 18% in the elderly [7], and 8%-15% in adults [8]. The Frykman classification of distal radius fractures is based on an odd and even numbering system. The even number is simply the addition of an ulnar styloid fracture to the previous odd number. Thus, Frykman type I is a transverse metaphyseal fracture and encompasses both Colles and Smith fractures. Type II is a type I plus an ulnar styloid fracture. Type III is a distal radius fracture that involves the radiocarpal joint, while type IV is a type III with an ulnar styloid fracture. Similarly, a type V Frykman fracture is a transverse fracture that involves the distal radioulnar joint, while type VI is a type V plus an ulnar styloid fracture. Finally, a type VII Frykman fracture is a comminuted fracture with the involvement of both the radiocarpal and radioulnar joints and a type VIII Frykman fracture is VII with a fracture of the ulnar styloid [5]. 

The epidemiology of these fractures changes by the age group (children and elderly are high-risk) and contributing factors. Trends show that in most age groups, women were at greater risk for a Frykman fracture than men. Additionally, in older populations, these injuries occur more frequently in patients with osteoporosis and can lead to the development of symptomatic post-traumatic arthritis. Overall, distal radius fractures are estimated to account for ~20% of all emergency department visits [8].

Typically, patients who suffer wrist trauma present to the ED and have x-rays to confirm their injuries. For displaced radius fractures, a hematoma block (HB) is typically performed and the fracture is reduced and splinted [9]. In most adults, intra-articular radius fractures require operative intervention within 10 days of the injury. There are a few different methods of operative intervention for treating distal radius fractures. Options include external fixation (EF), a volar locking plate (VLP), and Kirschner wire (K-wire). All forms of fixation provide adequate treatment of the fracture, and choosing the type of surgery should be based on the preferences of the surgeon, patient, and lifestyle choices [10]. Both operative and non-operative treatments as well as complications should be discussed with the patient. Some non-operative complications include nonunion, malunion, deformity, and tendon irritation or rupture. Additionally, surgical complications can include tenosynovitis from VLP, nerve injuries, infection, complex regional pain syndrome (CRPS), and posttraumatic arthritis [11]. High-trauma distal radius fractures can lead to compartment syndrome. This syndrome results from increased pressure near the site of fracture that obstructs blood supply to the tissues resulting in an orthopedic emergency that requires prompt intervention. Therefore, careful discussion with an orthopedic surgeon needs to occur as well as avoiding circumferential casting when there is a suspicion of compartment syndrome [12].

**Figure 2:**
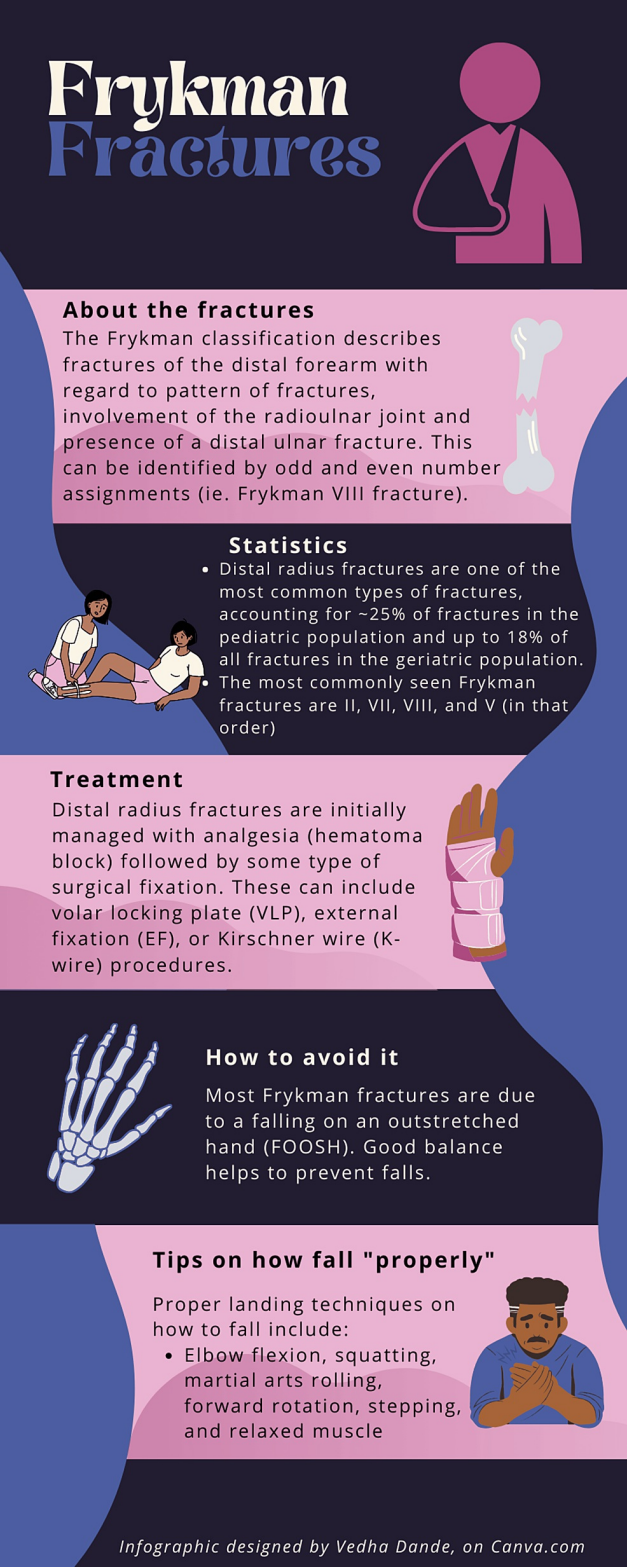
Infographic summarizing Frykman fractures and how to mitigate fall impact

There are preventative steps that can be taken to avoid wrist fractures, including safe landing strategies [13]. Examples of such strategies include elbow flexion, squatting, martial arts rolling, forward rotation, stepping, and relaxed muscle. Strategies like the listed six and education on how to fall properly may lead to fewer accidental falls and fractures in sports, and lifestyle (Figure 2).

## Conclusions

Frykman VIII is a type of fracture that relates to a break in the ulna styloid region of the wrist bone, as well as involving the radiocarpal and radioulnar joints. It is a fracture that typically results from trauma from a FOOSH mechanism. In the emergency department, it can be treated with a hematoma block, reduction, and splinting to decrease pain with a later surgical intervention. It is important to consider risk factors based on the epidemiology of distal radius fractures and educate patients on how to forward roll when falling to minimize a FOOSH-related wrist fracture.
